# Reevaluating progression and pathways following *Mycobacterium tuberculosis* infection within the spectrum of tuberculosis

**DOI:** 10.1073/pnas.2221186120

**Published:** 2023-11-14

**Authors:** Katherine C. Horton, Alexandra S. Richards, Jon C. Emery, Hanif Esmail, Rein M. G. J. Houben

**Affiliations:** ^a^Department of Infectious Disease Epidemiology, London School of Hygiene and Tropical Medicine, London WC1E 7HT, United Kingdom; ^b^Clinical Trials Unit, University College London, London WC1V 6LJ, United Kingdom

**Keywords:** tuberculosis, infection, disease, progression, incidence

## Abstract

Traditional understanding of the risk of progression from *Mycobacterium tuberculosis* (*Mtb*) infection to tuberculosis (TB) overlooks diverse presentations across a spectrum of disease. We developed a deterministic model of *Mtb* infection and minimal (pathological damage but not infectious), subclinical (infectious but no reported symptoms), and clinical (infectious and symptomatic) TB, informed by a rigorous evaluation of data from a systematic review of TB natural history. Using a Bayesian approach, we calibrated the model to data from historical cohorts that followed tuberculin-negative individuals to tuberculin conversion and TB, as well as data from cohorts that followed progression and regression between disease states, disease state prevalence ratios, disease duration, and mortality. We estimated incidence, pathways, and 10-y outcomes following *Mtb* infection for a simulated cohort. Then, 92.0% (95% uncertainty interval, UI, 91.4 to 92.5) of individuals self-cleared within 10 y of infection, while 7.9% (95% UI 7.4 to 8.5) progressed to TB. Of those, 68.6% (95% UI 65.4 to 72.0) developed infectious disease, and 33.2% (95% UI 29.9 to 36.4) progressed to clinical disease. While 98% of progression to minimal disease occurred within 2 y of infection, only 71% and 44% of subclinical and clinical disease, respectively, occurred within this period. Multiple progression pathways from infection were necessary to calibrate the model and 49.5% (95% UI 45.6 to 53.7) of those who developed infectious disease undulated between disease states. We identified heterogeneous pathways across disease states after *Mtb* infection, highlighting the need for clearly defined disease thresholds to inform more effective prevention and treatment efforts to end TB.

## Significance

Understanding of the risk of progression to tuberculosis (TB) after infection with *Mycobacterium tuberculosis* (*Mtb*) has traditionally relied on a binary distinction between infection and infectious, symptomatic disease. However, this advanced disease state is only one of many across a spectrum of disease presentations. We utilized mathematical modeling informed by an extensive systematic review of TB natural history to reevaluate progression and pathways following *Mtb* infection. We show the impact of different disease thresholds and highlight heterogeneous pathways through the spectrum of disease. These results update our understanding of progression risks and timelines in line with the spectrum of TB to guide more effective prevention, detection, and treatment efforts and avert morbidity and transmission to end TB.

Tuberculosis (TB) is a leading cause of morbidity and mortality and has substantial economic and social impacts worldwide (1). Despite growing acceptance that TB presents across a spectrum of disease states, defined by different pathological, bacteriological, and clinical thresholds (2–8), the pathways by which individuals progress from *Mycobacterium tuberculosis* (*Mtb*) infection and transition across thresholds are not well understood. Progression risks and timelines are founded instead on a conventional binary distinction between *Mtb* infection and infectious symptomatic TB. However, progression from infection to this advanced disease state is neither straightforward nor guaranteed.

*Mtb* infection is traditionally understood to confer a 5 to 10% lifetime risk of developing TB (1), with half of this risk occurring within 2 y of infection (9, 10). These axioms are typically understood to refer to infectious, usually symptomatic, disease (5, 11) and are often attributed to data from preventive chemotherapy trials (12) and Bacillus Calmette–Guérin vaccination trials (13). However, timelines from these studies are, at best, approximate, as none documents the time at which individuals were infected with *Mtb*. These studies also reflect the binary approach that ignores diverse presentations across the spectrum of disease and assumes unidirectional progression across a single threshold. Given the importance and wide reach of simplifying statements, the gaps in data for historical and more recent contributions to this canon are surprising.

Recognizing these limitations, and in line with the shift toward understanding TB across a spectrum of disease, there is a clear need to reevaluate our understanding of progression following *Mtb* infection. Intermediate disease states between *Mtb* infection and infectious symptomatic TB merit further attention. Infectious subclinical TB is as prevalent as infectious symptomatic disease globally (14) and likely contributes substantially to onward transmission (15–17). Pathological disease is also highly prevalent (18) and, even when noninfectious, can be severe. Focus on a single threshold of infectious symptomatic disease (19) ignores the potential contribution of intermediate disease states to morbidity, including post-TB lung disease, and onward transmission and may be a key reason why progress to reduce TB incidence and mortality remains slower than needed to meet targets (20, 21).

Expanded efforts under the End TB Strategy (20) have rightly renewed focus on disease prevention, including amongst those infected with *Mtb* (20, 22, 23), with research and innovation priorities that include the development of safe and effective treatment regimens for *Mtb* infections and postexposure prophylactic vaccines (24). Prevention strategies using these and other technologies are built on the binary approach, which informed the estimate of a quarter of the world carrying *Mtb* and at risk of progressing to TB disease (25), which is likely a substantial overestimate given new insights in the likelihood of individuals clearing their *Mtb* infection (26, 27). Development and implementation of novel prevention strategies, as well as resources for disease detection and treatment, require an updated understanding of the risk of progression following *Mtb* infection and subsequent pathways through the course of disease.

We therefore reevaluate progression and pathways following *Mtb* infection across the spectrum of disease, examining different thresholds of disease based on pathological, bacteriological, and clinical characteristics, and allowing for self-clearance of *Mtb* infection. Recent work quantified progression and regression between different disease states using an extensive systematic review of TB natural history (28) with mathematical modelling methods to simulate pathways for individuals with prevalent subclinical or clinical disease at baseline (29). We build on this work to incorporate dynamics relating to *Mtb* infection, allowing us to examine the full spectrum of infection and disease. With these developments, we estimate incidence, pathways, and 10-y outcomes following *Mtb* infection across different disease thresholds.

## Methods

Our analysis examines pathways from *Mtb* infection through minimal, subclinical, and clinical TB states, with clearance from infection, recovery from minimal disease, and mortality from clinical disease. We focus solely on pulmonary TB in adults and adolescents.

### Definitions.

Infection: Individuals with evidence of *Mtb* infection by an immunologic test in the absence of bacteriological evidence of TB or clinical signs or symptoms of TB (4, 5, 29, 30).

Cleared: Individuals who have effectively controlled or eliminated *Mtb* infection without developing TB and will not progress to TB in the absence of reinfection (5, 6, 26).

Recovered: Individuals who have effectively controlled or eliminated *Mtb* infection after developing TB and will not progress to TB in the absence of reinfection.

Minimal disease: Individuals with pathology prior to the onset of bacteriological evidence of TB, regardless of symptoms (29, 31).

Subclinical disease: Individuals with bacteriological evidence of TB who do not report symptoms of TB on screening (14, 29).

Clinical disease: Individuals with bacteriological evidence of TB with symptoms of TB (29).

Infectious disease: Bacteriologically positive disease; as such, it includes subclinical and clinical disease, but excludes minimal disease (29).

TB: Any state of minimal, subclinical, or clinical disease.

### Data Synthesis.

To synthesise evidence of progression following *Mtb* infection, we reviewed studies from a recent systematic review of TB natural history (28), as well as studies referenced by two recent papers on progression following *Mtb* infection (11, 32). We sought studies that followed cohorts of tuberculin-negative individuals to tuberculin conversion and then to TB (classified as minimal, subclinical, or clinical per definitions above). For inclusion, studies were required to document tuberculin conversion and report intervals between tuberculin testing, the number of individuals followed from tuberculin conversion for incident disease, intervals between disease screening following tuberculin conversion, the number of individuals who developed disease after tuberculin conversion, and the interval between tuberculin conversion and disease detection for those who developed disease.

We adjusted data to reflect uncertainty in the time of tuberculin conversion and disease onset, recognising that neither infection nor disease onset occurred at the point those developments were detected in these studies. To estimate time of infection, we sampled from a uniform distribution over each interval between the last negative tuberculin test and the first positive tuberculin test. To estimate time of disease onset, we sampled between the last disease-negative screening and the first disease-positive screening using a Cauchy distribution from time of tuberculin conversion informed by Poulsen (33). The population at risk was adjusted to remove individuals with incident disease at the appropriate time while also reflecting loss to follow-up as reported by each study. Data adjustments are discussed in greater detail in *SI Appendix*, *Data Adjustments*.

Data to inform transitions between minimal, subclinical, and clinical disease states were extracted from the same systematic review of TB natural history (28), as described in detail elsewhere (29). Data were also identified to inform mortality rate, duration of infectious TB, and prevalence ratios for minimal to infectious TB and subclinical to clinical TB (29).

### Model Development.

We expanded a model of the spectrum of TB (29) to incorporate *Mtb* infection. Model development relied on an iterative process through which we examined model structures with different pathways from infection to disease states to identify a structure that accurately reflected synthesised data with as simple a structure as possible. The model development process is discussed in *SI Appendix*, *Model Development*.

### Model Calibration.

**Fig. 1.**
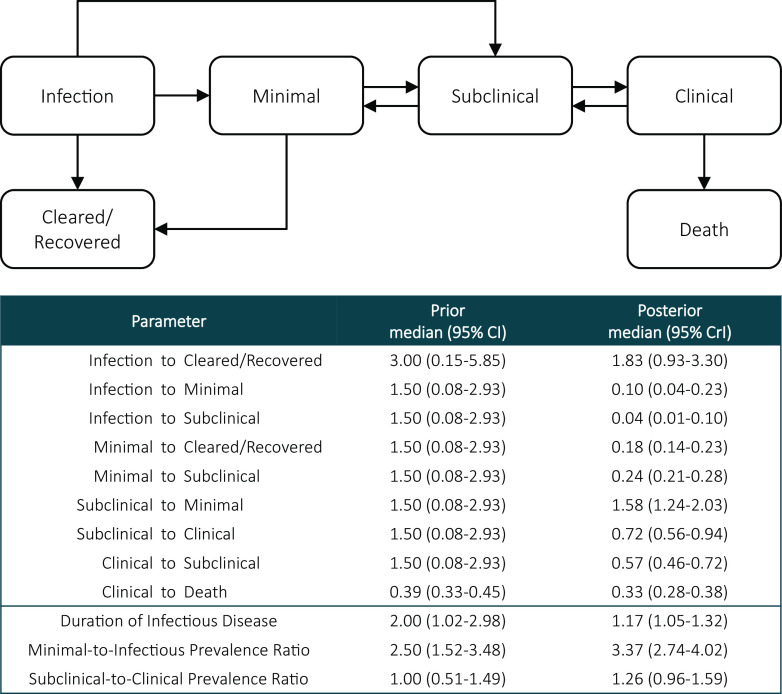
Model structure and prior and posterior parameters for pathways and progression following *Mtb* infection across the spectrum of TB.

Using a Bayesian approach, we calibrated the final model to data on progression following *Mtb* infection, as well as data on transitions between minimal, subclinical, and clinical disease states, mortality, duration of infectious TB, and prevalence ratios of minimal to infectious TB and subclinical to clinical TB. Data were weighted relative to the cohort size, regardless of the number of data points provided (29). All transition rates were assigned uninformed uniform priors [U(0.00,6.00) per year for clearance from infection; U(0.00,3.00) per year for all others] (29), with the exception of mortality rate [N(0.39,0.03) per year] (34). Informative priors were assigned to the duration of infectious TB [N(2.00,0.50) years] (35), the ratio of minimal to infectious TB prevalence [N(2.50,0.50)] (18), and the ratio of subclinical to clinical TB prevalence [N(1.00,0.25)] (14), as described elsewhere (29). Median values and 95% CIs for prior distributions are shown in Fig. 1.

Posterior estimates were calculated using a sequential Markov chain Monte-Carlo algorithm in LibBi (36) via R statistical software (37) with RBi (38) and rbi.helpers (39) packages. An adaptive process was used to run repeated chains of 1,000 iterations, adapting parameter values until the acceptance rate was between 20% and 30%. A further 150,000 iterations were then run and the initial adaptive iterations were discarded as burn-in. Convergence of the final 10,000 iterations was assessed visually. Posterior parameter estimates are presented as median values and credible intervals (CrIs).

### Progression Following Infection.

To quantify progression and pathways following *Mtb* infection, we simulated 1,000 cohorts of 100,000 individuals over a 10-y period from the time of *Mtb* infection with transitions at monthly intervals. Parameters were sampled independently for each individual at each time point in the simulation for each cohort to capture uncertainty.

Incidence estimates for minimal, subclinical, and clinical TB were generated in abbreviated models in which individuals exited the model after reaching the disease state of interest (*SI Appendix*, Fig. S2). As individuals can and do undulate between disease states, defining incidence is not straightforward. Here, we counted individuals as incident for each state on their first time entering that state following progression from a less advanced state. We report median and 95% uncertainty intervals (UIs) for annual and cumulative incidence over a 10-y period following *Mtb* infection.

### Pathways Following Infection.

Pathways through model states over a 10-y period following *Mtb* infection are reported. Individuals were considered to have experienced “undulation” if they progressed to any disease state, regressed from that disease state, and then progressed again to the same or a more advanced disease state (i.e., changed trajectory at least twice). Median and 95% UIs are reported for proportions of individuals following specific pathways.

Monthly transitions were also categorised to report a dominant disease state for each individual for each 12-mo period following *Mtb* infection. If, during a 12-mo period, an individual spent nine or more months in a single state, that state is reported for the 12-mo period. If, during a 12-mo period, an individual spent less than 9 mo in a single state or transitioned between states three or more times, the state is reported as “transitional” for the 12-mo period (29).

## Results

### Data Synthesis.

To inform progression from *Mtb* infection to TB, we screened 54 studies of TB natural history: Forty-nine from the systematic review (28) and five from other studies (11, 32). We reviewed the full text of 18 potentially relevant studies, excluded 15 studies (13, 33, 40–58), and identified three studies for inclusion (35, 59–61). Excluded studies are described in *SI Appendix*, Table S1.

Two studies followed individuals from tuberculin conversion to minimal disease. Daniels (59) describes follow-up of 722 tuberculin-negative nursing students who were enrolled between 1935 and 1943 in London, United Kingdom. Tuberculin conversion was detected by annual screening in 248 participants, who were then followed annually for up to 5 y. Twenty-seven individuals developed TB disease, defined via radiography per Prophit committee standards (59). Madsen (60) describes follow-up of 1,099 tuberculin-negative medical and high school students enrolled between 1934 and 1936 in Copenhagen, Denmark. Tuberculin conversion was detected by approximately annual screening in 208 participants, who were then followed approximately annually for up to 6 y. Fifty-two individuals developed TB, defined as demonstrable chest x-ray changes following tuberculin conversion (60).

One study followed individuals from tuberculin conversion to infectious disease. The National Tuberculosis Institute describes a longitudinal study of TB natural history conducted between 1961 and 1968 in Bangalore, India (35, 61). Four surveys were conducted, with 18-mo intervals between the first and second and second and third and a 24-mo interval between the third and fourth. Tuberculin testing was performed at each survey for previously tuberculin-negative individuals; disease screening was performed for previously tuberculin-positive individuals. Tuberculin conversion was detected in 1,538 participants. Forty-two individuals developed TB, defined as chest x-ray positive and sputum culture-positive, regardless of symptoms. Data from this study are included due to the limited evidence available elsewhere, with recognition that investigators’ decision to withhold effective TB treatment from study participants would now be viewed as highly unethical. See *SI Appendix*, *Data Synthesis* for more detail.

As in previous analysis (29), 22 studies informed transition rates between minimal, subclinical, and clinical disease states (35, 50, 58, 61–94). Data were also gathered to inform mortality rate (34), duration of infectious TB (95), and prevalence ratios for minimal to infectious TB (14) and subclinical to clinical TB (18). These data are described in detail elsewhere (29).

### Model Development.

The final model structure reflects heterogeneous progression following *Mtb* infection, with pathways for progression from infection to either minimal or subclinical disease, and allows clearance from infection (Fig. 1). The model allows progression and regression between minimal, subclinical, and clinical disease states, with recovery from the minimal state and TB-associated mortality from the clinical state only (29).

### Model Calibration.

Medians and 95% CrIs for posterior parameter distributions are shown in Fig. 1. Median rates for progression from infection to minimal disease and progression from infection to subclinical disease were 0.10 (95% CrI 0.04 to 0.23) and 0.04 (95% CrI 0.01 to 0.10) per year, respectively. The median clearance rate was 1.83 (95% CrI 0.93 to 3.30) per year from infection. Remaining posterior parameter rates are consistent with previous research (29).

Posterior calibrations and weighted calibration targets are shown in *SI Appendix*, Figs. S3–S5.

### Progression Following Infection.

**Fig. 2.**
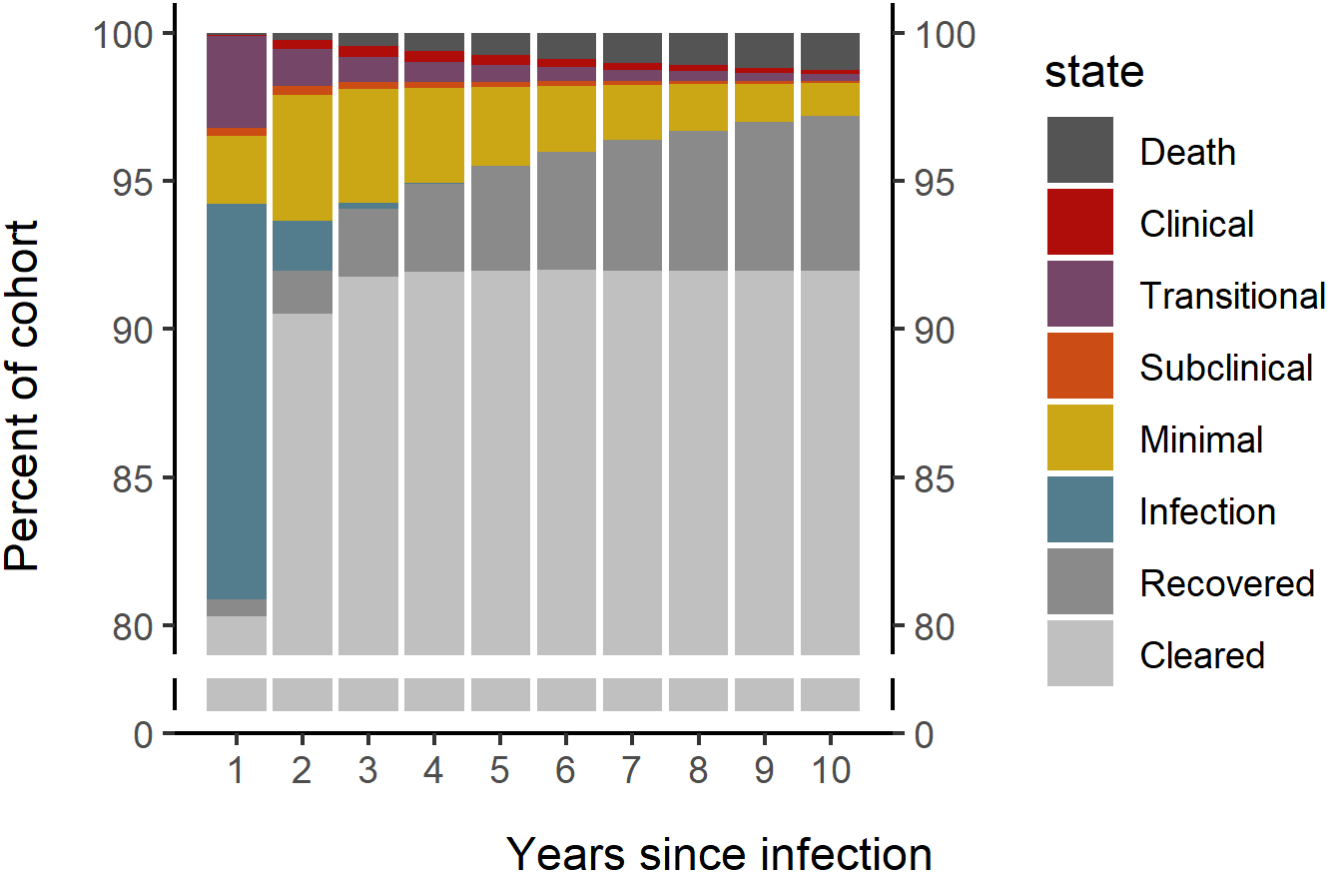
Median distribution of simulated individuals by dominant disease state for each year over 10 y following *Mtb* infection.

Over a 10-y period following *Mtb* infection, 92.0% (95% UI 91.4 to 92.5) of simulated individuals cleared infection without progressing to TB, with 90.5% (95% UI 89.9 to 91.1) clearing infection within the first 2 y (Fig. 2).

**Fig. 3.**
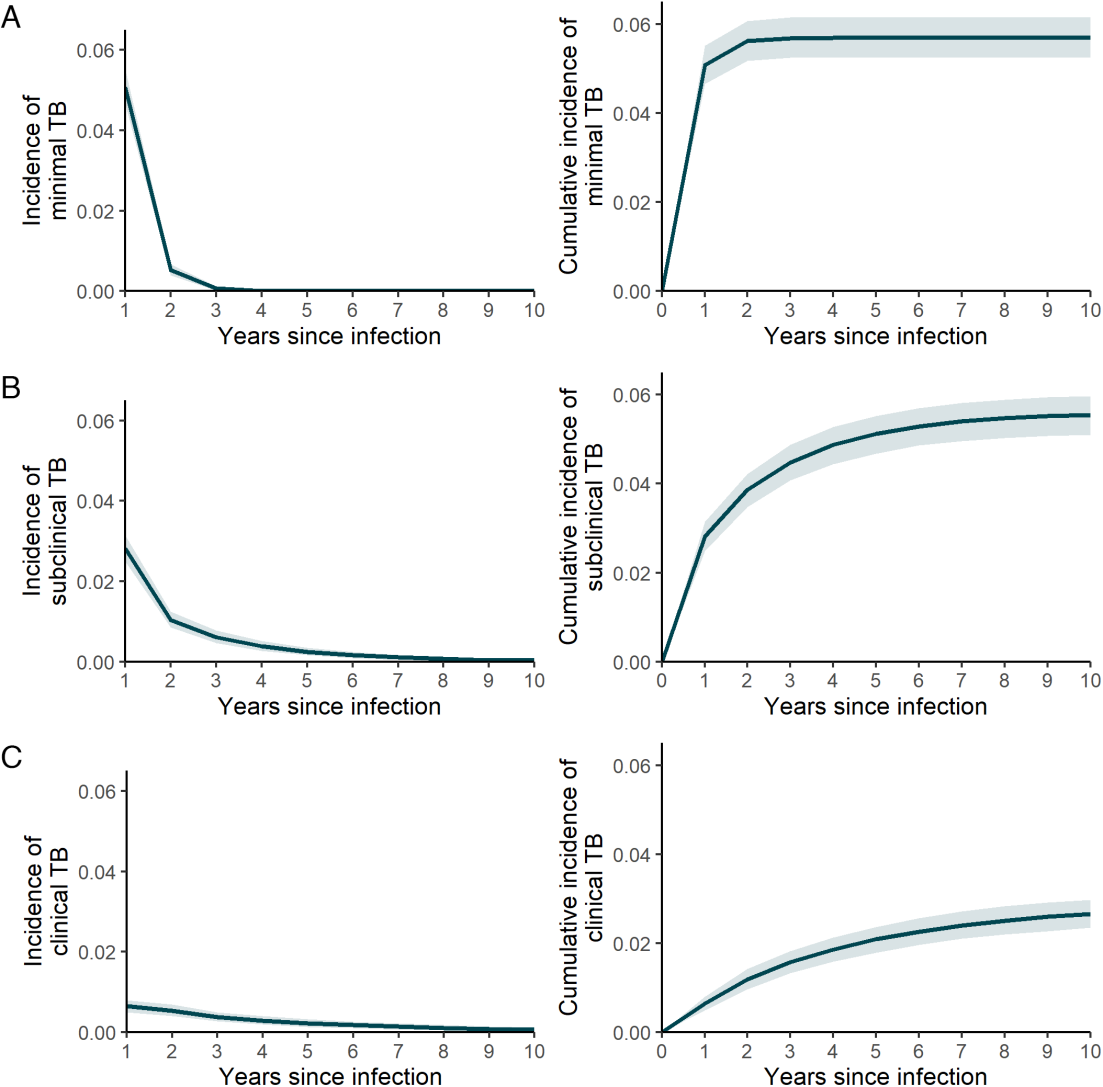
Annual (*Left*) and cumulative (*Right*) incidence for minimal (*A*), subclinical (*B*), and clinical (*C*) TB disease over a 10-y period following *Mtb* infection. Lines and shaded areas show medians and 95% UIs for calibrated model. Points and error bars show medians and 95% CIs for calibration targets for minimal and subclinical TB disease.

Cumulative incidence of TB was 7.9% (95% UI 7.4 to 8.5) at 2 y and 8.0% (95% UI 7.5 to 8.6) at 10 y in this simulated cohort. At 2 y postinfection, cumulative incidence of minimal, subclinical, and clinical disease was 5.6% (95% UI 5.2 to 6.1), 3.9% (95% UI 3.5 to 4.2), and 1.2% (95% UI 1.0 to 1.4), respectively. Further incidence over the period from 3 to 10 y gave a 10-y cumulative incidence of 5.7% (95% UI 5.3 to 6.2), 5.5% (95% UI 5.1 to 6.0), and 2.7 (95% UI 2.4 to 3.0), respectively, for minimal, subclinical, and clinical disease. Annual and cumulative incidence of minimal, subclinical, and clinical TB in this simulated cohort over a 10-y period following *Mtb* infection are shown in Fig. 3.

### Pathways Following Infection.

**Fig. 4.**
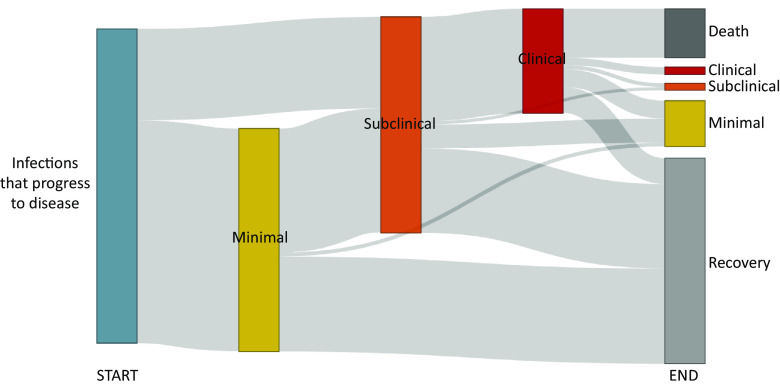
Sankey diagram showing broad pathways over a 10-y period following *Mtb* infection for simulated individuals who developed TB. Start represents time of infection for simulated individuals who progress to TB. End shows the distribution of those simulated individuals across states after 10 y. Neither undulation nor detailed timelines for transitions between states is shown.

Of the simulated individuals who developed TB, 70.9% (95% UI 67.8 to 74.2) progressed from infection to minimal disease, while 29.1% (95% UI 25.8 to 32.2) progressed directly from infection to subclinical disease (Fig. 4). Then, 31.4% (95% UI 28.0 to 34.6) of simulated individuals developed minimal disease and did not progress to infectious disease within 10 y of infection. In total, 68.6% (95% UI 65.4 to 72.0) of simulated individuals who progressed to disease developed subclinical disease. Then, 35.5% (95% UI 32.1 to 38.9) of simulated individuals developed subclinical disease but did not progress further to clinical disease within 10 y of infection, while 33.2% (95% UI 29.9 to 36.4) progressed to clinical disease. Of the simulated individuals who developed TB, 15.6% (95% UI 13.3 to 18.2) died from TB-associated mortality in this untreated cohort, and 65.1% (95% UI 61.9 to 68.2) recovered within 10 y of infection.

Of the simulated individuals who developed TB, 56.4% (95% UI 53.1 to 59.6) progressed and then regressed towards recovery, either recovering or remaining in a less advanced disease state at the end of the 10-y period. Another 33.9% (95% UI 30.9 to 37.2) of the simulated individuals who developed TB—49.5% (95% UI 45.6 to 53.7) of those who developed infectious disease—undulated between states (i.e., progressed, regressed, then progressed again) in their course of disease. These individuals transitioned between disease states a median 5 (95% UI 3 to 11) times. The final 8.2% (95% UI 6.3 to 10.2) of simulated individuals who developed TB disease progressed with no regression during the 10-y period following infection.

## Discussion

Our rigorous evaluation of historical data and application within a Bayesian modelling framework allows us to differentiate progression risk and pathways following *Mtb* infection by different disease thresholds across the spectrum of TB. Over a 10-y period following *Mtb* infection, one in 10 simulated individuals progressed to TB. Of those, two-thirds developed infectious disease, half of which progressed to clinical disease. Timeframes for incidence varied substantially between disease thresholds. While nearly all progression to minimal disease and most progression to subclinical disease occurred within 2 y of infection, half of all progression to clinical disease occurred later in the course of disease. Heterogeneous progression pathways from *Mtb* infection were necessary to calibrate the model, and pathways following infection were diverse with half of those who developed infectious disease undulating between disease states.

Our findings show that different thresholds of disease have different implications for understanding pathways following *Mtb* infection. The traditional threshold of infectious symptomatic disease recognises only a third of our simulated cohort who progressed beyond *Mtb* infection and after those individuals had progressed through less advanced disease states. A lower threshold acknowledging any infectious disease, regardless of reported symptoms, recognised another third of the cohort in addition to those with infectious, symptomatic disease. This encompassed all those who contribute to *Mtb* transmission (15, 96). As nearly half of individuals who developed subclinical TB went on to develop clinical TB, a threshold of infectious disease has potential individual benefit through the opportunity to avert further morbidity and possible mortality, as well as population benefit from the interruption of further transmission. Yet, this threshold still omits a third of individuals who progressed from *Mtb* infection in our study. Though these individuals are considered noninfectious, more than half went on to develop infectious disease, so intervention at this threshold could avert not only more severe morbidity, possibly including post-TB lung disease, but also truly prevent future transmission.

Our findings emphasise a need for caution and greater attention to definitions when considering broad statements about timelines of disease risk. Our findings are consistent with the lower end of the conventional lifetime risk of progression to TB when considering a threshold of minimal or subclinical disease, but our estimated 10-y cumulative incidence of clinical disease is well below that axiom. Our results dispute assertions that the vast majority of infectious, symptomatic TB develops less than 2 y after *Mtb* infection (50). We found that just under half of progression to clinical disease occurs during this period, though nearly all progression to minimal disease and most progression to subclinical disease occurred within this time.

There is much heterogeneity in pathways following *Mtb* infection. In our model development, we found that data on progression following *Mtb* infection cannot be explained without allowing multiple progression paths, including rapid progression to infectious disease and the opportunity to clear *Mtb* infection. Our simplified pathways reflecting progression to minimal disease and progression to subclinical disease may be considered in line with traditional considerations of “rapid” and “slow” progression following infection. The proportion of self-clearance in our model reflects the inclusion of clearance as an output and the indirect influence of data used for calibration; no informative prior was assigned for the rate of self-clearance. While there are no direct data to inform either the total amount or the distribution of self-clearance over time, it is notable that the resulting median 90.4% self-clearance in our simulated cohort is consistent with data-driven estimates from others (27). We have also highlighted that complex pathways through the spectrum of TB are common. Pathways reflecting only progression through the course of disease were rare, occurring for only a tenth of individuals who developed disease. More than half of individuals experienced at least some regression towards recovery in their course of disease, while half of those who developed infectious disease undulated between disease states. Among these individuals, the number of transitions between disease states had a wide range. Transitional periods of frequent transitions were also common, particularly in the first years after infection.

Our evaluation of potential data sources to inform this work was rigorous. We identified three studies which documented timed tuberculin conversions for 2,000 individuals (as a proxy for *Mtb* infection) and approximate time of disease onset, as well as information on the population at risk which allowed us to calculate incidence risks over time. This methodology overcomes limitations of other data sources, including some frequently cited (12, 13), that, most often, do not report timing of tuberculin conversion but may also have unclear disease endpoints or lack data on the underlying population of tuberculin converters at risk of developing disease. Pairing historic (prechemotherapy) cohort data on progression from infection and transitions within the spectrum of disease with contemporary (postchemotherapy) data on the distribution of prevalence across the disease spectrum is a particular strength of our model.

Our work has several limitations. We focus solely on pulmonary TB and cannot comment on extrapulmonary disease. Our model is calibrated to separate data for each transition rather than data following trajectories across the spectrum of disease. As such, each transition is independent of any prior disease state or pathway, while in reality previous disease state episodes likely have some influence on future pathways. Our assessment of undulation focuses on progression and regression between states and therefore ignores fluctuations that do not result in transitions between defined states. We also do not consider risk factors for progression from *Mtb* infection, such as age, sex, or HIV infection, due to the lack of disaggregated reporting in included studies. Further research is needed to examine whether our findings are consistent across these subgroups.

Our data-driven modelling work quantifies differences in progression across different thresholds of disease following *Mtb* infection and highlights heterogeneous pathways through the spectrum of TB. These results update our understanding of progression risks and timelines in line with the spectrum of disease to guide more effective prevention, detection, and treatment efforts and ultimately avert morbidity and transmission in order to end TB.

## Supplementary Material

Appendix 01 (PDF)

## Data Availability

Replication data and analyses scripts are available on GitHub (https://github.com/ERC188TBornotTB/Inf-Dis) (97).
