# Supplementary material for: Reevaluating progression and pathways following Mycobacterium tuberculosis infection within the spectrum of tuberculosis
Source: Proc Natl Acad Sci U S A. 2023 Nov 14;120(47):e2221186120. doi: 10.1073/pnas.2221186120 (PMC10666121; doi:10.1073/pnas.2221186120)
Supplement: Supplementary file 1 — Appendix 01 (PDF) [file pnas.2221186120.sapp.pdf]

Supplemental Information

I. Data Synthesis

The 15 studies excluded after full text review are reported in Supplemental Table 1 with the reason for exclusion for each.

Supplemental Table 1: Reasons for exclusion of potentially relevant studies

Study	Reason for exclusion
Beeuwkes H, Hahn RG, Putnam P. A Survey of Persons Exposed to Tuberculosis in the Household1, The Necessity for Prolonged Observation of Contacts. American Review of Tuberculosis. 1942;45(2):165-93.	Time to disease onset is measured from contact or exposure rather than known tuberculin conversion.
Borgen L, Meyer SN, Refsum E. Mass photofluorography, tuberculin testing, and BCG vaccination in the district of Aker (Norway) 1947-49. Acta Tuberculosea Scandinavica. 1951;25(4):327-55.<br>Borgen L, Meyer SN. Mass investigation by photofluorography: An illustration of the value of the method in combating tuberculosis. Acta tuberculosea et pneumologica Scandinavica. 1951;25:288-302.	Time of tuberculin conversion is not reported; disease onset is reported by initial tuberculin test results.
Ferebee SH, Mount FW, Murray FJ, Livesay VT. A controlled trial of isoniazid prophylaxis in mental institutions. American Review of Respiratory Disease. 1963;88(2):161-75.	Time of tuberculin conversion is not reported for most participants. Two participants are reported to have converted within 12 months of enrolment, but time to disease onset for these individuals is not reported.
Ferebee SH, Mount FW. Tuberculosis morbidity in a controlled trial of the prophylactic use of isoniazid among household contacts. American Review of Respiratory Disease. 1962;85(4):490-510.	Time of tuberculin conversion is not reported.
Frimodt-Møller J, Thomas J, Parthasarathy R. Observations on the protective effect of BCG vaccination in a South Indian rural population. Bulletin of the World Health Organization. 1964;30(4):545.<br>Frimodt-Møller J. A community-wide tuberculosis survey in a South Indian rural population, 1950-55. Bulletin of the World Health Organization. 1960;22(1-2):61.	Time of tuberculin conversion is not reported; disease onset is reported by initial tuberculin test results.
Gedde-Dahl T. Tuberculous infection in the light of tuberculin matriculation. American Journal of Epidemiology. 1952;56(2):139-214.	Intervals between tuberculin testing are unknown (performed at “suitable intervals”), and intervals between chest x-ray following tuberculin conversion are inconsistent.
Hart PA. BCG and vole bacillus vaccines in the prevention of tuberculosis in adolescence and early adult life. Third report to the Medical Research Council by their Tuberculosis	Time of tuberculin conversion is not reported; disease onset is reported by initial tuberculin test results.

Vaccines Clinical Trials Committee. BMJ. 1963;2:973-8.<br>Hart PA. BCG and vole bacillus vaccines in the prevention of tuberculosis in adolescents. First (Progress) report to the Medical Research Council by their tuberculosis vaccines clinical trials committee. British Medical Journal. 1956:413-27.<br>Hart PA. BCG and vole bacillus vaccines in the prevention of tuberculosis in adolescents. Second report to the Medical Research Council by their Tuberculosis Vaccines Clinical Trials Committee. BMJ. 1959;2:379-96.	
Israel HL, Hetherington H, Ord JG. A study of tuberculosis among students of nursing. Journal of the American Medical Association. 1941;117(10):839-44.	Disease onset is not reported by tuberculin status.
Okada K, Onozaki I, Yamada N, Yoshiyama T, Miura T, Saint S, et al. Epidemiological impact of mass tuberculosis screening: a 2-year follow-up after a national prevalence survey. The International journal of tuberculosis and lung disease. 2012;16(12):1619-24.	Time of tuberculin conversion is not reported.
Poulsen A. Some clinical features of tuberculosis. 1. Incubation period. Acta tuberculosea Scandinavica. 1950;24(3-4):311-46.<br>Poulsen A. Some clinical features of tuberculosis. Acta tuberculosea Scandinavica. 1957;33(1-2):37.	Intervals between tuberculin testing are uncertain and inconsistent across the population ("the majority of the population has been submitted to systematic tuberculin tests… at intervals of one or two years") and not all "converters" had prior evidence of a negative tuberculin reaction.
Rubinstein H, Kotschnowa I. Beginn und entwicklung der lungentuberkulose beim erwachsenen. Acta Medica URSS. 1940;3(3):250-65.	Time of tuberculin conversion is not reported; disease onset is reported by initial chest x-ray results.
Sikand B, Narain R, Mathur G. Incidence of TB as Judged by Re-surveys A Study of Delhi Police By. 1959.	Time of tuberculin conversion is not reported; tuberculin testing was only performed at enrolment.
Stýblo K, Dankova D, Drapela J, Galliova J, Ježek Z, Křivánek J, et al. Epidemiological and clinical study of tuberculosis in the district of Kolin, Czechoslovakia: report for the first 4 years of the study (1961-64). Bulletin of the World Health Organization. 1967;37(6):819.	Time of tuberculin conversion is not reported; disease onset is reported by initial chest x-ray results.
Sutherland I. The ten-year incidence of clinical tuberculosis following 'conversion' in 2,550 individuals aged 14 to 19 at time of conversion. The Hague, The Netherlands: Tuberculosis Surveillance Research Unit, 1968.	Intervals between disease screening are inconsistent with inclusion of routine passive diagnoses. Authors assume a uniform distribution of disease onset between "last normal" and "first abnormal" chest radiograph, which is inconsistent with empiric evidence (see Data Adjustments).
Wallgren A. The time-table of tuberculosis. Tubercle. 1948;29(11):245-51.	No description of the study methodology underlying reported time between exposure and disease onset could be found.

Data from the longitudinal study of TB natural history conducted by the National Tuberculosis Institute in Bangalore, India, are included as evidence of progression following *Mtb* infection (1, 2). Authors of this study, conducted between 1961 and 1968, state that “no organized antituberculosis treatment was available to the people of the area during the entire study period” (1). However, they also report that “[d]uring the second survey and the early part of the third, with a view to obtaining better cooperation, one month's supply of isoniazid tablets was issued to persons in whom pulmonary tuberculosis was diagnosed” (1). This limited course of isoniazid would have had little effect on disease progression and may have contributed to the emergence of drug resistant strains of *Mtb*. Authors’ acknowledgement of this limited provision implies that investigators had knowledge of and access to isoniazid, yet it appears no efforts were made to provide an effective regimen of the drug to study participants outside the scope of improving “cooperation”. We have decided to include data from this research, which would now be considered highly unethical, because we were unable to identify any other data sources that could provide comparable data, and we feel not using the data would make the burden placed on study participants even more onerous.

II. Data Adjustments

After extracting data from three included studies describing progression from *Mtb* infection to TB, we adjusted those data to reflect uncertainty in the time of tuberculin conversion and disease onset, recognising that neither infection nor disease onset occur at the point those developments are detected in these studies.

For studies reporting progression from TST conversion to minimal disease, we first reduced the number of reported cases by 25% to acknowledge that some pathological anomalies detected by chest x-ray may not have been attributable to TB (3).

For all studies, to estimate time of infection, we sampled from a uniform distribution over the interval between the last negative tuberculin test and the first positive tuberculin test.

For all studies, to estimate time of disease onset, we sampled between the last disease negative screening and the first disease positive screening. The distribution from which we sampled was informed by data from Poulsen (1957) (4) on the proportion of incident cases of TB over time following infection, shown in Supplemental Table *2*. Various distributions were examined, and a Cauchy distribution (location=2.0600957, shape=0.8282028) was selected as the best fit to the data. This distribution was aligned with the previously sampled time of tuberculin conversion and truncated to sample within the interval between the last negative disease screen and the first positive disease screen.

Supplemental Table 2: Proportion and cumulative proportion of incident cases of TB by years since infection per Poulsen (1957)

Years since infection	Proportion of incident cases	Cumulative proportion of incident cases
1	0.17	0.17
2	0.35	0.52
3	0.18	0.70
4	0.17	0.87
5	0.06	0.93
6	0.04	0.97
8	0.01	0.98
10	0.02	1.00

We adjusted the number of tuberculin converters at risk of disease to remove individuals with incident disease at the appropriate time while also reflecting loss to follow-up as reported by each study. We note that no loss to follow-up was reported in manuscripts describing the National Tuberculosis Institute study in Bangalore, India, which risks underestimating progression to subclinical disease in later years following infection. Sensitivity analyses (not shown) indicate that model estimates are robust to minor changes to individual data sources, so potential underestimation of long-term progression in this study is expected to have little impact on posterior transition parameters.

Raw and adjusted data are shown in Supplemental Table 3 and unweighted adjusted incidence is shown in Supplemental Table 4.

Supplemental Table 3: Raw and adjusted data showing years since infection, number of events reported, and population at risk for each study

Study	Raw data			Adjusted data		
	Year	Event	At risk	Year	Events	At risk
Daniels 1944 (5)	1	19	248	1	5	248
	2	4	198	2	10	212
	3	3	134	3	3	128
	4	1	58	4	1	58
	5	0	16	5	0	16
Madsen 1942 (6)	1	41	208	1	32	208
	2	9	162	2	7	171
	3	2	109	3	1	111
	4	0	63	4	0	64
	5	0	26	5	0	26
	6	0	16	6	0	16
NTI 1974 (1), Krishnamurthy 1976 (2)	1.5	19	674	1	17	674
				2	3	657
	3	2	-	3	2	654
	5	4	-	4	1	652
				5	0	651
NTI 1974 (1), Krishnamurthy 1976 (2)	1.5	7	451	1	7	451
				2	2	444

	3.5	2	-	3	1	442
				4	0	441
NTI 1974 (1), Krishnamurthy 1976 (2)	2	8	413	1	6	413
				2	2	407

Supplemental Table 4: Adjusted data showing years since infection, number of events reported, population at risk, and unweighted incidence of minimal or subclinical TB for each study

Study	Year	Events	At risk	Incidence of minimal TB (%)
Daniels 1944 (5)	1	5	248	2.0 (0.7-4.6)
	2	10	212	4.7 (2.3-8.5)
	3	3	128	2.3 (0.5-6.7)
	4	1	58	1.7 (0.0-9.2)
	5	0	16	0.0 (0.0-20.6)
Madsen 1942 (6)	1	32	208	15.4 (10.8-21)
	2	7	171	4.1 (1.7-8.3)
	3	1	111	0.9 (0.0-4.9)
	4	0	64	0.0 (0.0-5.6)
	5	0	26	0.0 (0.0-13.2)
	6	0	16	0.0 (0.0-20.6)
Study	Year	Events	At risk	Incidence of subclinical TB (%)
NTI 1974 (1), Krishnamurthy 1976 (2)	1	17	674	2.5 (1.5-4.0)
	2	3	657	0.5 (0.1-1.3)
	3	2	654	0.3 (0.0-1.1)
	4	1	652	0.2 (0.0-0.9)
	5	0	651	0.0 (0.0-0.6)
NTI 1974 (1), Krishnamurthy 1976 (2)	1	7	451	1.6 (0.6-3.2)
	2	2	444	0.5 (0.1-1.6)
	3	1	442	0.2 (0.0-1.3)
	4	0	441	0.0 (0.0-0.8)
NTI 1974 (1), Krishnamurthy 1976 (2)	1	6	413	1.5 (0.5-3.1)
	2	2	407	0.5 (0.1-1.8)

III. Model Development

The model structure relating *Mtb* infection to TB states was developed through an iterative process. In total, we examined 4 potential model structures defining progression from *Mtb* infection in different ways (Supplemental Figure 1). Model A allowed only progression from infection to minimal disease. Model B allowed direct progression from infection to subclinical disease in addition to progression from infection to minimal disease. Model C allowed progression to subclinical disease, progression to minimal disease, and indirect progression to minimal disease via an intermediate state. Model D allowed direct progression from infection to clinical disease, in addition to progression from infection to minimal disease and progression from infection to subclinical disease.

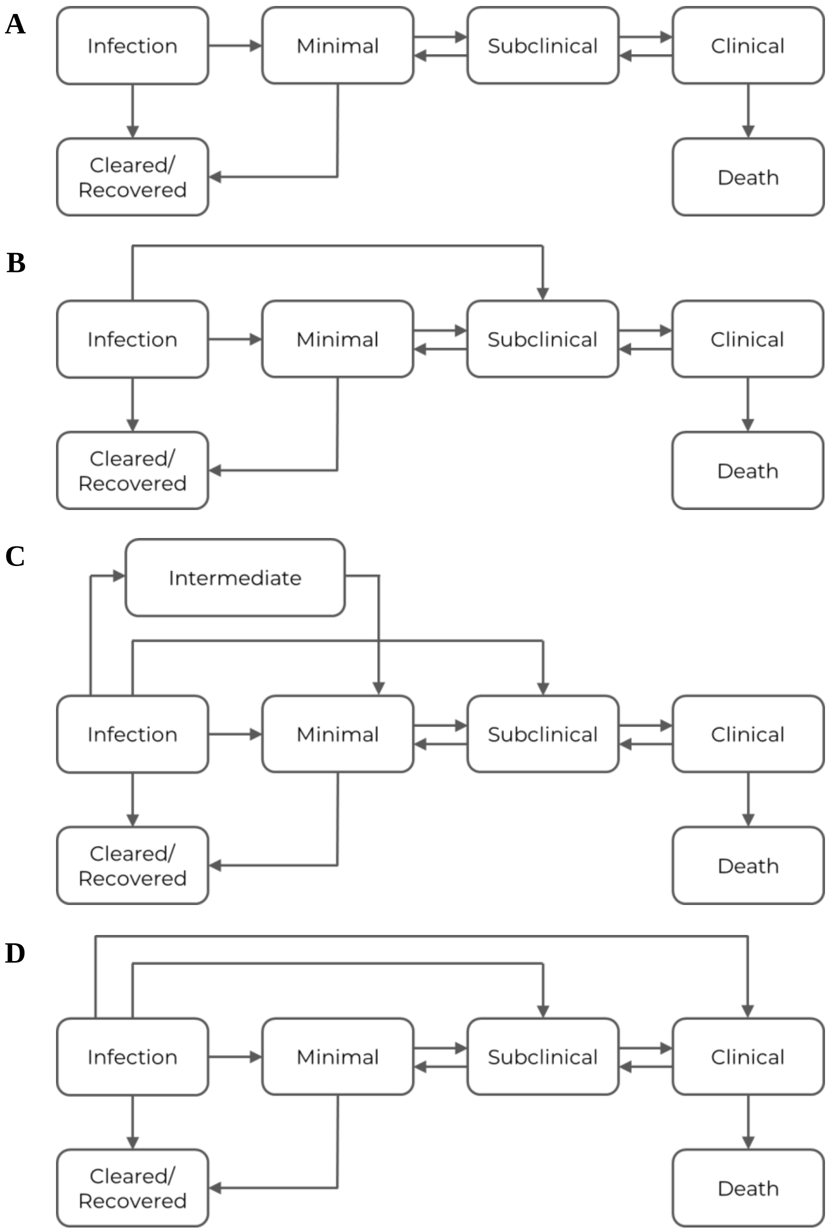

Supplemental Figure 1: Potential model structures examined during the model development process

Each model structure was calibrated following methods described in the main text Methods and Supplemental Information Model Calibration. Models were evaluated based on visual inspection of trace and correlation plots, visual inspection of posterior calibrations to progression from infection to minimal disease and from infection to subclinical disease, and deviance information criteria (DIC). Calibrations for each model are shown in

Supplemental Table 5.

Supplemental Table 5: Model development calibrations. Error bars show calibration targets, and lines and shading show median and 95% uncertainty intervals for calibrated model.

	Calibration to minimal disease	Calibration to subclinical disease	Trace plots	DIC
**A**	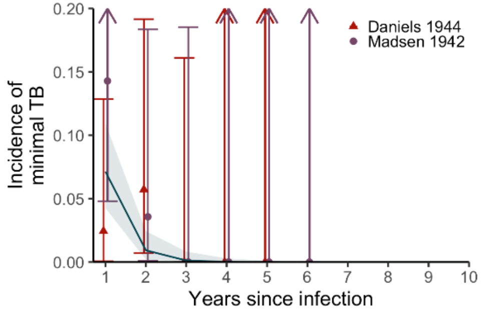	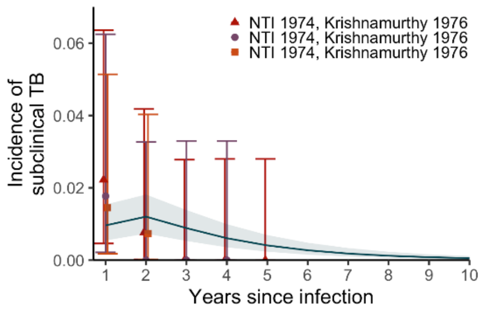	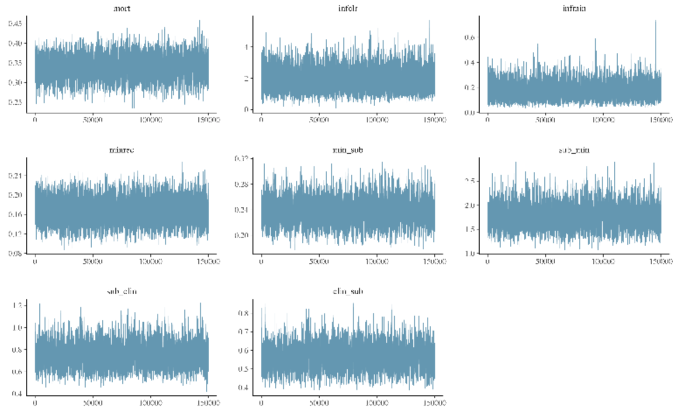	657.4
**B**	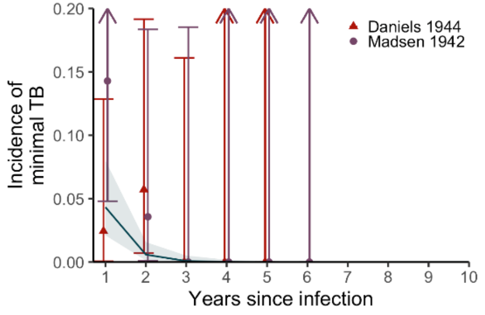	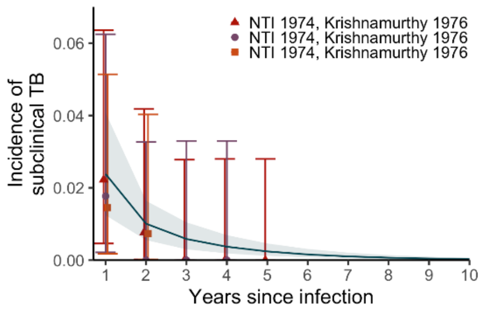	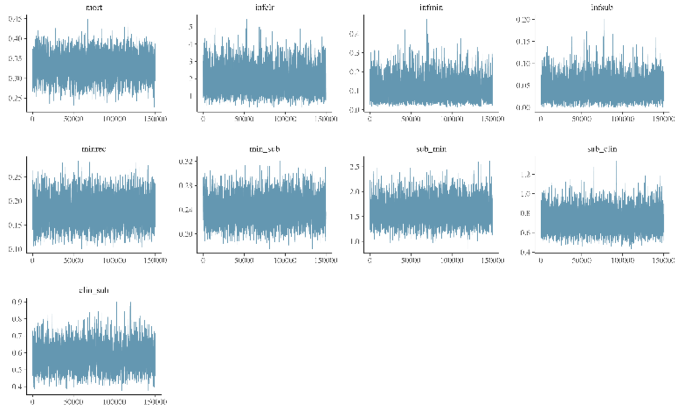	663.8

<table>
<tr><th></th><th>Calibration to minimal disease</th><th>Calibration to subclinical disease</th><th>Trace plots</th><th>DIC</th></tr>
<tr><td>C</td><td>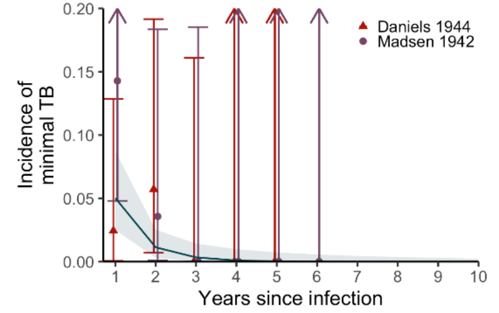
</td><td>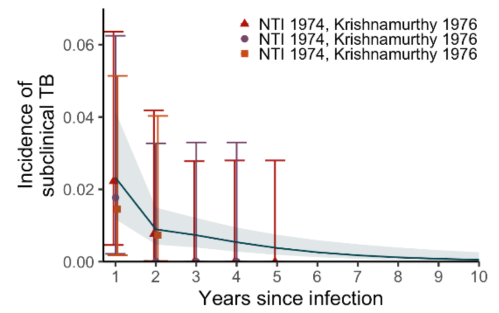
</td><td>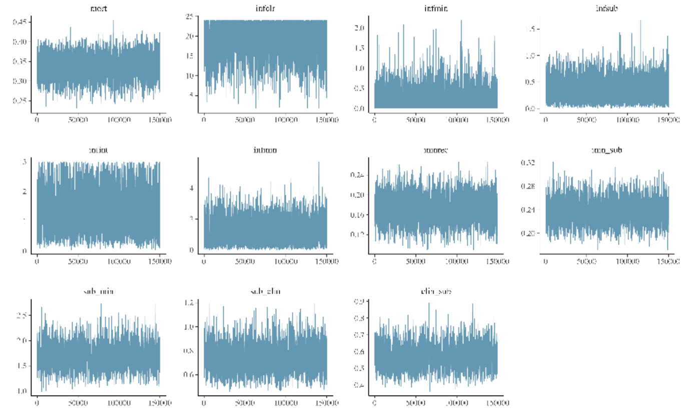</td><td>657.5</td></tr>
<tr><td>D</td><td>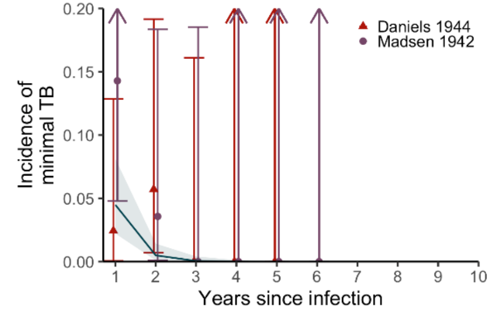
</td><td>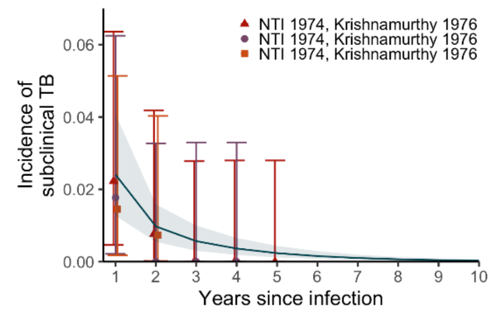
</td><td>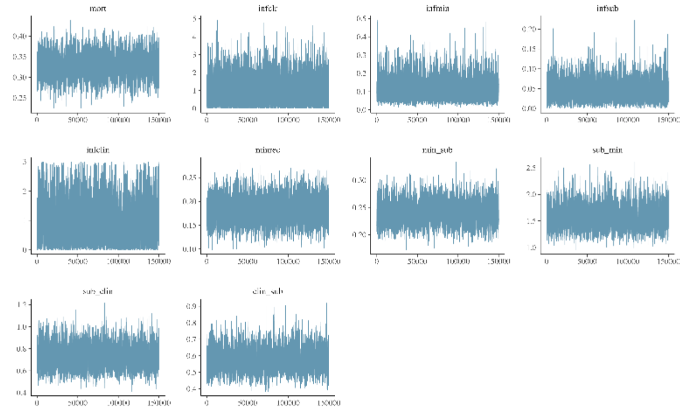</td><td>665.1</td></tr>
</table>

For Model A, the calibrated incidence of minimal disease was considered reasonable, but the shape of the calibrated incidence of subclinical disease deviated from the shape of the data for this calibration target. For Model B, the shape of the calibrated incidence of subclinical disease was improved, relative to Model A. Trace plots for models C and D suggest these model structures are not consistent with empirical data. Therefore Model B was selected as the most parsimonious for the main model analysis.

IV. Model Calibration

The equations used to describe the main model are as follows:

$$\frac{dI}{dt} = -(infclr + infmin + infsub) * I$$

$$\frac{dM}{dt} = infmin * I - (minrec + minsub) * M + submin * S$$

$$\frac{dS}{dt} = infsub * I + minsub * M - (submin + subclin) * S + clinsub * C$$

$$\frac{dC}{dt} = subclin * S - (clinsub + clinmort) * C$$

where I, M, S, and C refer to infection, minimal, subclinical, and clinical states, respectively, and remaining terms represent transition rates with *infclr* from infection to cleared/recovered, *infmin* from infection to minimal, *minrec* from minimal to cleared/recovered, *minsub* from minimal to subclinical, *submin* from subclinical to minimal, *infsub* from infection to subclinical, *subclin* from subclinical to clinical, *clinsub* from clinical to subclinical, and *clinmort* from clinical to death.

Appropriate sub-structures from this model were used in the calibration process to align with the data informing calibration targets for different transitions (3). Model structures used to calculate incidence of minimal, subclinical, and clinical disease are shown in Supplemental Figure 2.

A

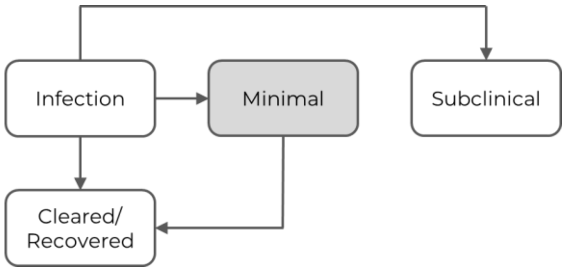

B

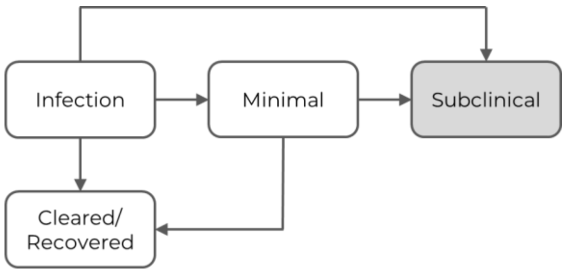

C

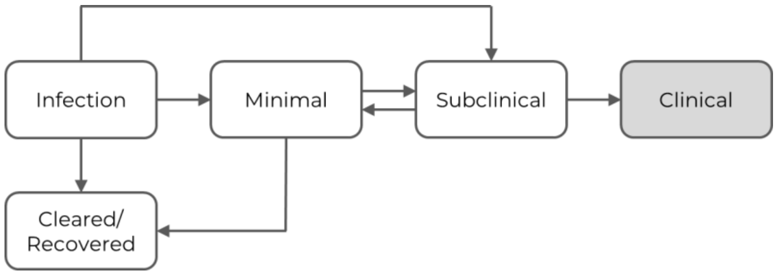

Supplemental Figure 2: Model structure to quantify incidence of minimal (A), subclinical (B), and clinical (C) TB

Posterior calibrations and weighted calibration targets for progression from infection to minimal and subclinical disease states are shown in Supplemental Figure 3, transitions between minimal, subclinical, and clinical disease states in Supplemental Figure 4, and additional contemporary data points in Supplemental Figure 5.

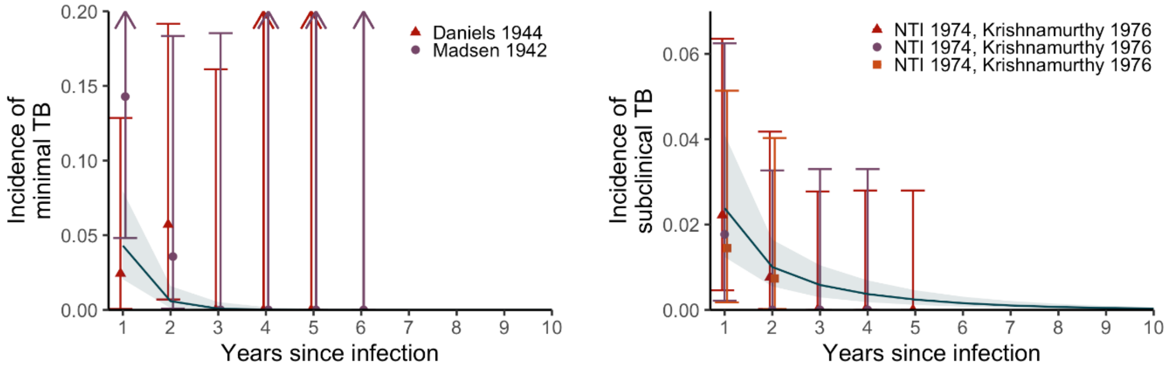

Supplemental Figure 3: Model calibration for progression from infection to minimal (left) and subclinical (right) disease states

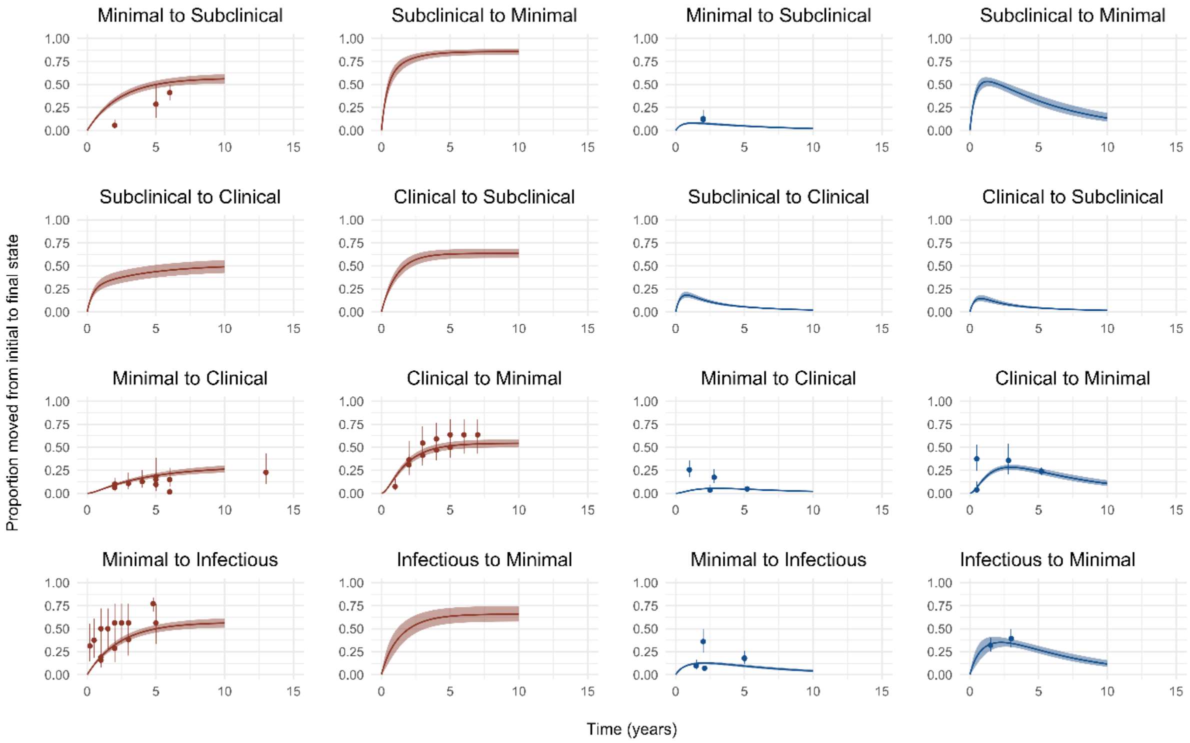

Supplemental Figure 4: Model calibration for transitions between minimal, subclinical, and clinical disease states

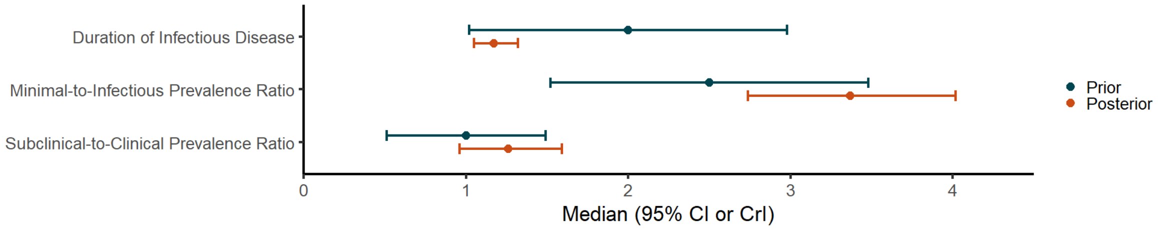

Supplemental Figure 5: Model calibration for additional contemporary data points

Trace plots and correlation plots for each parameter over the 150,000 iterations of the calibrated model are shown in Supplemental Figure 6 and Supplemental Figure 7, respectively.

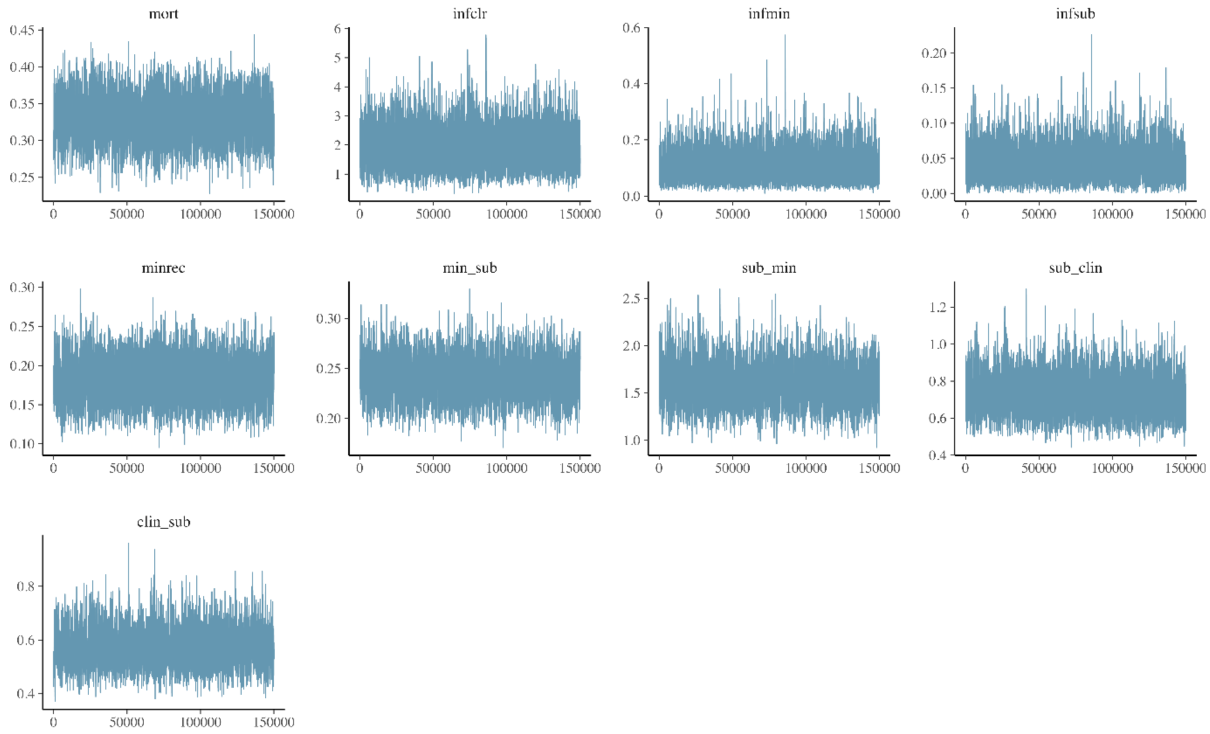

Supplemental Figure 6: Trace plots for calibrated model

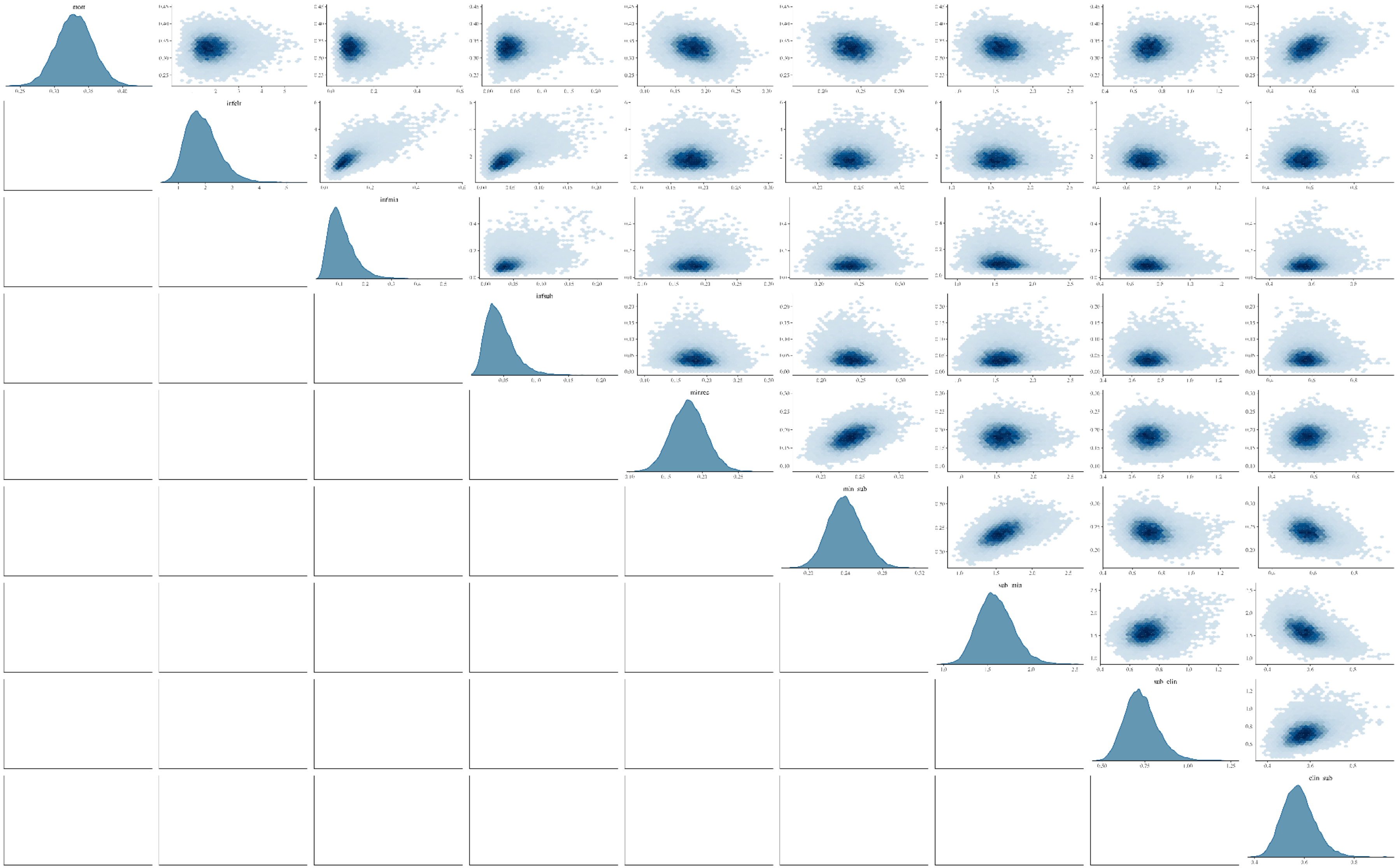

Supplemental Figure 7: Correlation plots for calibrated model

V. Sensitivity Analysis

We conducted sensitivity analyses to explore uncertainty in the disease states represented by NTI data due to the lack of reporting of any clinical signs or symptoms. We examined three scenarios with different degrees of clinical representation among cases, classifying cases as either 0%, 50%, or 100% clinical. Calibrations are shown in Supplemental Figure 8 and Supplemental Table 6.

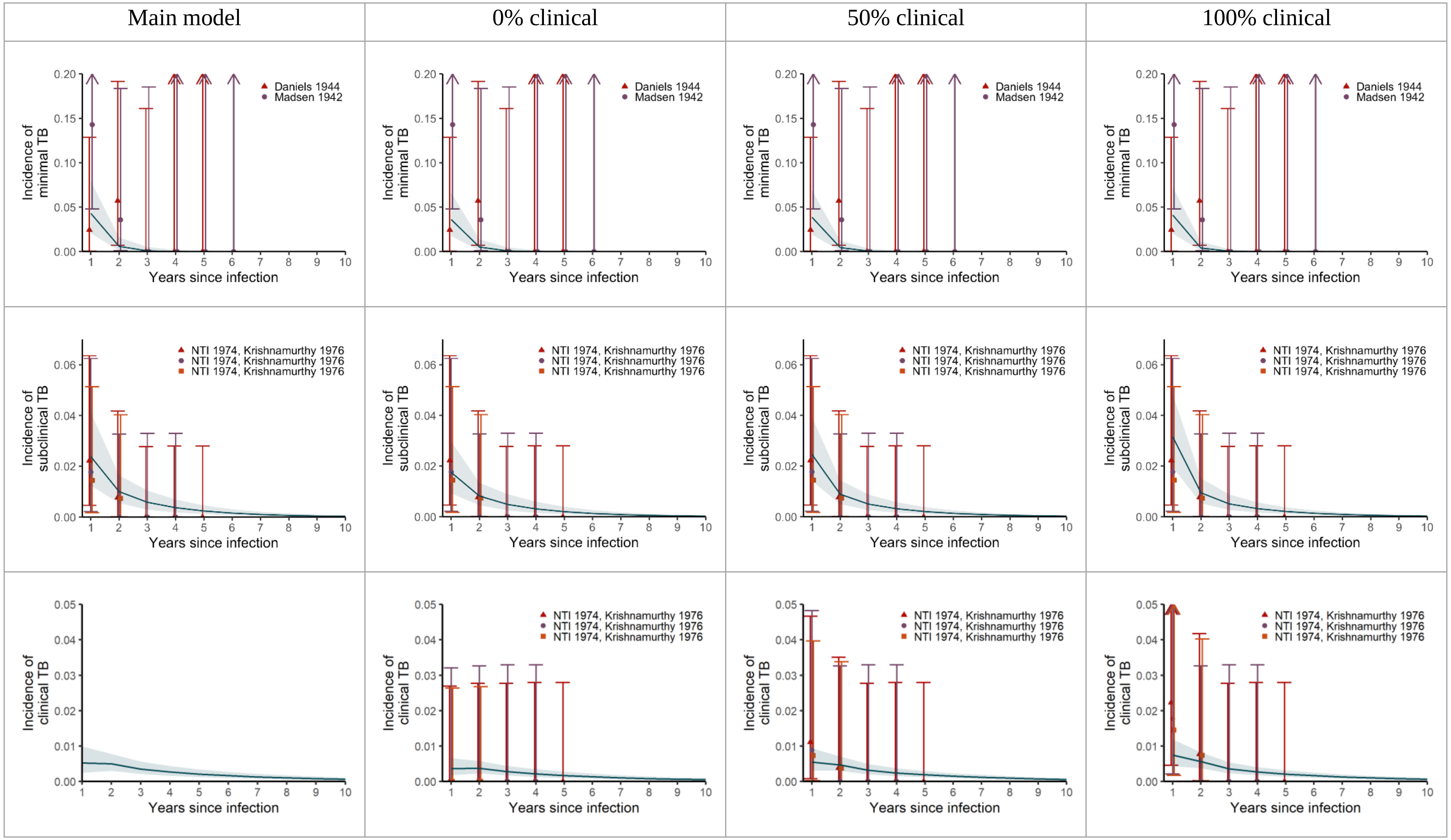

Supplemental Figure 8: Sensitivity analysis calibrations for different classifications of NTI data

Supplemental Table 6: Posterior parameters for different classifications of NTI data

Parameter	Main model	0% clinical	50% clinical	100% clinical
Infection to Cleared/Recovered	1.83 (0.94-3.30)	1.82 (0.93-3.23)	1.99 (1.08-3.44)	2.14 (1.22-3.61)
Infection to Minimal	0.10 (0.04-0.23)	0.08 (0.03-0.19)	0.09 (0.04-0.21)	0.11 (0.04-0.23)
Infection to Subclinical	0.04 (0.01-0.10)	0.03 (0.01-0.07)	0.05 (0.02-0.10)	0.07 (0.03-0.13)
Minimal to Cleared/Recovered	0.18 (0.14-0.23)	0.18 (0.14-0.23)	0.18 (0.14-0.23)	0.18 (0.13-0.23)
Minimal to Subclinical	0.24 (0.20-0.28)	0.24 (0.21-0.28)	0.24 (0.21-0.28)	0.24 (0.20-0.27)
Subclinical to Minimal	1.58 (1.22-2.05)	1.59 (1.24-2.03)	1.61 (1.26-2.07)	1.61 (1.25-2.08)
Subclinical to Clinical	0.72 (0.56-0.95)	0.69 (0.54-0.90)	0.72 (0.57-0.94)	0.76 (0.59-0.99)
Clinical to Subclinical	0.58 (0.46-0.73)	0.56 (0.45-0.70)	0.57 (0.46-0.72)	0.58 (0.47-0.74)
Clinical to Death	0.33 (0.28-0.38)	0.33 (0.28-0.38)	0.33 (0.28-0.38)	0.33 (0.28-0.39)
Duration of Infectious Disease	1.17 (1.05-1.32)	1.17 (1.05-1.32)	1.17 (1.04-1.32)	1.16 (1.04-1.31)
Minimal-to-Infectious Prevalence Ratio	3.36 (2.75-4.03)	3.46 (2.82-4.12)	3.32 (2.71-3.96)	3.21 (2.62-3.86)
Subclinical-to-Clinical Prevalence Ratio	1.26 (0.95-1.59)	1.29 (0.99-1.63)	1.25 (0.95-1.58)	1.22 (0.93-1.55)

VI. References

1. National Tuberculosis Institute, Tuberculosis in a rural population of South India: a five-year epidemiological study. *Bulletin of the World Health Organization* **51**, 473 (1974).

2. V. V. Krishnamurthy, S. S. Nair, G. D. Gothi, A. K. Chakraborty, Incidence of Tuberculosis among newly infected population and in relation to the duration of infected status. *Indian Journal of Tuberculosis* **23** (1976).

3. A. S. Richards *et al.*, Quantifying progression and regression across the spectrum of pulmonary tuberculosis: a data synthesis study. *The Lancet Global Health* **11**, E684-E692 (2023).

4. A. Poulsen, Some clinical features of tuberculosis. *Acta Tuberculosea Scandinavica* **33**, 37 (1957).

5. M. Daniels, Primary tuberculous infection in nurses: manifestations and prognosis. *The Lancet* **244**, 165-170 (1944).

6. T. Madsen, J. Holm, K. A. Jensen, Studies on the epidemiology of tuberculosis in Denmark. *Acta Tuberculosea Scandinavica* (1942).
